# A comprehensive phylogenomic framework for cycads (Cycadales)

**DOI:** 10.3897/phytokeys.275.194283

**Published:** 2026-05-22

**Authors:** Michael Calonje, James A. R. Clugston, Mario Coiro

**Affiliations:** 1 Montgomery Botanical Center, Coral Gables, Florida, USA SENCKENBERG – Leibniz Institution for Biodiversity and Earth System Research (SGN), Senckenberg Research Institute and Natural History Museum Frankfurt Frankfurt am Main Germany; 2 Hawkesbury Institute for the Environment, Western Sydney University, Locked Bag 1797, Penrith, NSW 2751, Australia Montgomery Botanical Center Coral Gables United States of America; 3 SENCKENBERG – Leibniz Institution for Biodiversity and Earth System Research (SGN), Senckenberg Research Institute and Natural History Museum Frankfurt, Frankfurt am Main, Germany Hawkesbury Institute for the Environment, Western Sydney University Penrith Australia

**Keywords:** Cycadales, divergence times, gene concordance factors, molecular dating, phylogenomics, site concordance factors, timetree, transcriptomics

## Abstract

Here we present a time-calibrated phylogeny of 346 cycad accessions, covering ≈86% of the 380 accepted species, across all 10 extant genera, inferred from 1,409 single-copy nuclear loci (411,345 amino acid sites) derived from transcriptome and genome data. The maximum likelihood phylogeny was inferred using a partitioned analysis, with branch support assessed via ultrafast bootstrap (UFBoot2), and concordance evaluated using gene concordance factors (gCF) and site concordance factors (sCF), representing gene- and site-level support, respectively. Divergence times were estimated using penalized likelihood (TreePL) with 12 calibration constraints, and 95% confidence intervals were derived from 100 gene-wise bootstrap replicates. Bootstrap support is high (70% of nodes ≥95%), but gene concordance factors are low (median gCF = 3.2%), a pattern consistent with limited phylogenetic signal per locus rather than strong support for alternative topologies. Across all 10 genera, the phylogram recovered a strongly supported generic backbone, confirmed the monophyly of all genera, and provides the first broadly accessible phylogenomic framework for interpreting cycad taxonomy, intergeneric relationships, and evolutionary history. Herein, we provide the phylogram, timetree, all 1,409 gene trees, the concatenated alignment with partition definitions, and associated support, confidence-interval, and calibration data.

## Introduction

The cycads (Cycadales) are among the most ancient lineages of extant seed plants, with a fossil record extending to the late Carboniferous and Permian (*ca*. 300 Ma; Coiro et al. 2023). Cycads comprise 380 recognized species in two families and 10 genera (Calonje et al. 2026): Cycadaceae (*Cycas* L.) and Zamiaceae (*Bowenia* Hook. ex Hook.f., *Ceratozamia* Brongn., *Dioon* Lindl., *Encephalartos* Lehm., *Lepidozamia* Regel, *Macrozamia* Miq., *Microcycas* (Miq.) A.DC., *Stangeria* T.Moore, and *Zamia* L.). Although cycads have long been characterized as “living fossils”, molecular dating studies have suggested that much of the extant species diversity within cycad genera originated during relatively recent Neogene radiations (Nagalingum et al. 2011; Condamine et al. 2015), although crown age estimates vary among studies depending on calibration strategy and taxon sampling. Consistent with this view, recent studies of cycad leaf-form diversity have shown that morphological diversity has been dynamic and expanding rather than static, with the fossil record revealing leaf forms absent among extant species (Coiro and Seyfullah 2024). Similar questions apply to reproductive morphology, where fossil evidence suggests that cycad strobili in deep time were more diverse than those of extant taxa (Elgorriaga and Atkinson 2023). Resolving these questions about the tempo and mode of morphological evolution will require a densely sampled, openly accessible species-level phylogeny against which hypotheses of vegetative and reproductive trait evolution can be explicitly mapped. In recent years, the availability of cycad transcriptomes has become an excellent resource for most cycad species through a combination of genomic and phylotranscriptomic studies (Habib et al. 2022; Liu et al. 2022; Habib et al. 2023; Lindstrom et al. 2024; Habib et al. 2025; Liu et al. 2026). Taken together, these datasets enable dense sampling across Cycadales and support phylogenomic analyses spanning all 10 genera. Liu et al. (2022) inferred a phylogeny of 339 cycad species from 1,170 low-copy nuclear genes as part of a broader study of the *Cycas* genome and the evolution of seed plants, with divergence times estimated from a 100-gene subset. However, the species-level phylogeny was presented only as a summary radial chronogram, and machine-readable tree files have not been deposited in a public repository. Other molecular phylogenies of cycads have relied on limited numbers of markers or taxa (Salas-Leiva et al. 2013) or have focused on individual genera (Habib et al. 2022, 2023; Lindstrom et al. 2024; Gutiérrez-Ortega et al. 2024; Liu et al. 2026). Despite recent growth in cycad molecular resources, an openly accessible species-level phylogenomic framework remains lacking.

Such a framework is needed to place taxonomic, biogeographic, and comparative analyses in an explicit evolutionary context, to identify species groups that warrant denser population-level sampling within genera, and to support conservation analyses that depend on species-level relationships. Here, we use publicly available cycad transcriptomes together with the reference genome of *Cycas panzhihuaensis* to build that framework for Cycadales, sampling 346 cycad accessions representing 326 accepted species (≈86% of the 380 currently accepted species in the World List of Cycads; Calonje et al. 2026) across all 10 genera, based on 1,409 single-copy nuclear loci identified from transcriptome-derived proteomes. We provide a maximum-likelihood phylogram with branch support and concordance factor data, a time-calibrated chronogram with 95% confidence intervals for all node ages, and the underlying gene trees and concatenated alignment. Together these resources provide the phylogenomic foundation needed to address outstanding questions in cycad systematics, biogeography, and comparative biology at the species level. All data are archived in Zenodo at https://doi.org/10.5281/zenodo.20074063 and linked to the World List of Cycads, a curated online taxonomic reference for accepted names and species-level cycad information.

## Methods

### Taxon sampling

The World List of Cycads (Calonje et al. 2026) recognizes 380 accepted cycad species in 10 genera. Our ingroup dataset comprises 346 cycad accessions spanning all 10 genera and both families; together, these represent approximately 86% of accepted cycad species. *Ginkgo biloba* was included as the outgroup, giving a total of 347 terminals in the phylogenetic analyses. These accessions include named species, infraspecific taxa, and provisionally identified samples, some of which may represent undescribed or as yet unresolved taxa. In a small number of cases, multiple accessions from different individuals of the same species were retained as separate terminals. Protein sequences for *Cycas panzhihuaensis* were obtained from its reference genome (Liu et al. 2022), whereas all other taxa were represented by *de novo* transcriptome assemblies derived from publicly available RNA-seq data deposited in the NCBI Sequence Read Archive (SRA; see Suppl. material 1 for accession numbers). Because some species are represented by more than one accession and the dataset also includes infraspecific taxa, the 346 cycad accessions correspond to approximately 326 unique accepted species. Taxonomy and nomenclature follow the World List of Cycads (Calonje et al. 2026); where names associated with the source transcriptomes have since been corrected or placed in synonymy, the current accepted name is used in the tree, with the original name recorded in Suppl. material 1. As a result, the same accepted species name may appear on more than one terminal in the deposited trees when multiple accessions were retained or when distinct source identifications were brought into synonymy under a single accepted name.

### Transcriptome assembly and processing

Paired-end Illumina RNA-seq reads were downloaded from the NCBI Sequence Read Archive for all sampled cycad accessions. *De novo* transcriptome assembly was performed with Trinity v2.15.1 (Grabherr et al. 2011), with integrated quality trimming via Trimmomatic with the default paired-end settings (ILLUMINACLIP:TruSeq3-PE.fa:2:30:10; SLIDINGWINDOW:4:5; LEADING:5; TRAILING:5; MINLEN:25). To reduce redundancy, the longest isoform per Trinity gene was retained using a bundled Trinity utility script. Protein-coding regions were then predicted using TransDecoder (Haas et al. 2013), which identifies open reading frames (ORFs) of at least 100 amino acids and applies a machine-learning classifier to distinguish coding from non-coding sequences. Where multiple ORFs were predicted per transcript, the longest protein was then selected and retained.

### Ortholog identification

Orthologous gene families were identified from predicted protein sequences for all sampled accessions (346 cycad accessions + *Ginkgo biloba*) using OrthoFinder v2.5.4 (Emms and Kelly 2019). OrthoFinder was run with DIAMOND as the underlying protein similarity-search engine in ultra-sensitive mode. A relaxed single-copy ortholog strategy was employed: loci were retained if present in at least 75% of cycad accessions (≥260 of 346), with no more than 10% of accessions permitted to carry a second copy. This approach yielded 1,409 loci with an average taxon occupancy of 94.9%, a 12-fold increase over strictly single-copy orthologs (115 loci), while maintaining high orthology confidence.

### Sequence alignment, gene trees, and paralog pruning

Each of the 1,409 orthogroups was independently aligned at the amino acid level using MAFFT v7.490 (Katoh and Standley 2013) with automatic algorithm selection. Poorly aligned and gap-rich regions were removed with trimAl v1.4 (Capella-Gutiérrez et al. 2009), using the automated1 heuristic. An individual gene tree was then inferred for each locus using IQ-TREE v3.0.1 (Minh et al. 2020b; Wong et al. 2025), with automatic model selection. For loci containing multiple copies per species, a phylogenetically informed pruning step was applied to the individual gene trees: for each species with multiple copies, the copy with the shortest average distance to all other accessions was retained, preferentially selecting orthologs over paralogs. The corresponding sequences of pruned copies were then removed from each individual alignment before concatenation.

### Phylogenetic inference

A partitioned maximum likelihood analysis was conducted using IQ-TREE, treating each of the 1,409 loci as an independent partition with its own best-fit substitution model selected by ModelFinder (Kalyaanamoorthy et al. 2017). The concatenated alignment comprised 411,345 amino acid sites. The most frequent best-fit models were Q.MAMMAL+G4 (41.5% of partitions), VT+R3 (8.2%), and JTT+G4 (4.5%). Branch support was assessed using 1,000 ultrafast bootstrap replicates (UFBoot2; Hoang et al. 2018), and the tree was rooted using *Ginkgo biloba* as the outgroup during inference using the -o flag.

### Concordance factors

Two complementary measures of topological concordance were estimated in IQ-TREE for gene concordance factors (gCF), which quantify the percentage of the 1,409 individual gene trees that recover each bipartition in the species tree. Site concordance factors (sCF) were then used to quantify the percentage of decisive alignment sites supporting each branch via likelihood-based quartet sampling (1,000 quartets per branch), under the partition-specific best-fit substitution models from the partitioned analysis (Mo et al. 2023). Together with bootstrap values, these metrics provide a multi-layered assessment of branch support that distinguishes statistical sampling support from genomic concordance.

### Divergence time estimation

Divergence times were estimated using TreePL v1.0 (Smith and O’Meara 2012), using a penalized likelihood method. *Ginkgo biloba* was pruned prior to dating, as its extreme divergence from cycads destabilizes rate smoothing across the tree. The optimal smoothing parameter was determined by cross-validation. Twelve age constraints were applied as minimum and maximum bounds (Table 1). Five deep-node calibrations were derived from the fossil-calibrated total-evidence analysis of Coiro et al. (2023), while seven genus-level calibrations were applied as secondary constraints using the 95% confidence intervals from recent molecular dating studies (Habib et al. 2022; Habib et al. 2023; Gutiérrez-Ortega et al. 2024; Lindstrom et al. 2024; Habib et al. 2025).

**Table 1.** Age constraints used for divergence time estimation. Calibrations 1–5 are fossil-informed node ages from the total-evidence analysis of Coiro et al. (2023). Calibrations 6–12 are secondary calibrations derived from confidence intervals of recent molecular dating studies. All ages are in millions of years (Ma), applied as minimum and maximum constraints on the most recent common ancestor (MRCA) of the specified taxa pair.

#	Node	Taxa pair (MRCA)	Min (Ma)	Max (Ma)	Reference
1	Cycadales crown	*Cycas taitungensis* + *Zamia integrifolia*	291.2	358.9	Coiro et al. (2023)
2	Zamiaceae crown	*Dioon spinulosum* + *Zamia integrifolia*	159.8	236.3	Coiro et al. (2023)
3	*Stangeria*–*Zamia*	*Stangeria eriopus* + *Zamia integrifolia*	118.8	187.3	Coiro et al. (2023)
4	*Lepidozamia*–*Macrozamia*	*Lepidozamia hopei* + *Macrozamia fraseri*	63.2	111.5	Coiro et al. (2023)
5	*Zamia*–*Microcycas*	*Zamia integrifolia* + *Microcycas calocoma*	65.7	119.3	Coiro et al. (2023)
6	*Ceratozamia* crown	*Ceratozamia matudae* + *C. alvarezii*	12.8	35.9	Habib et al. (2023)
7	*Zamia* crown	*Zamia integrifolia* + *Z. amazonum*	18.4	32.6	Lindstrom et al. (2024)
8	*Encephalartos* crown	*Encephalartos humilis* + *E. aemulans*	25.5	26.8	Habib et al. (2025)
9	*Macrozamia* crown	*Macrozamia fraseri* + *M. lucida*	11.5	28.8	Habib et al. (2022); Coiro et al. (2023)
10	*Lepidozamia* crown	*Lepidozamia hopei* + *L. peroffskyana*	15.7	34.3	Coiro et al. (2023)
11	*Dioon* crown	*Dioon spinulosum* + *D. califanoi*	19.4	56.6	Gutiérrez-Ortega et al. (2024); Coiro et al. (2023)
12	*Cycas* crown	*Cycas taitungensis* + *C. aculeata*	18.1	40.1	Coiro et al. (2023)

### Bootstrap confidence intervals

Uncertainty in divergence time estimates was quantified through a gene-wise bootstrap resampling approach. In each of 100 replicates, with the 1,409 loci being resampled with replacement, branch lengths were re-optimized on the fixed ML topology in IQ-TREE, and the resulting trees were dated with TreePL under the same calibration scheme. Node ages from all 100 replicates were compiled, and 95% confidence intervals were calculated as the 2.5^th^ and 97.5^th^ percentiles for all 345 internal nodes.

### Website dissemination

An interactive visualization of the produced phylogram and timetree has been implemented on the World List of Cycads website (www.cycadlist.org) using phylotree.js (Shank et al. 2018) with custom JavaScript controls for switching between phylogram and timetree views and for toggling branch-support annotations.

## Results

### Phylogenetic inference and support

**Figure 1.**
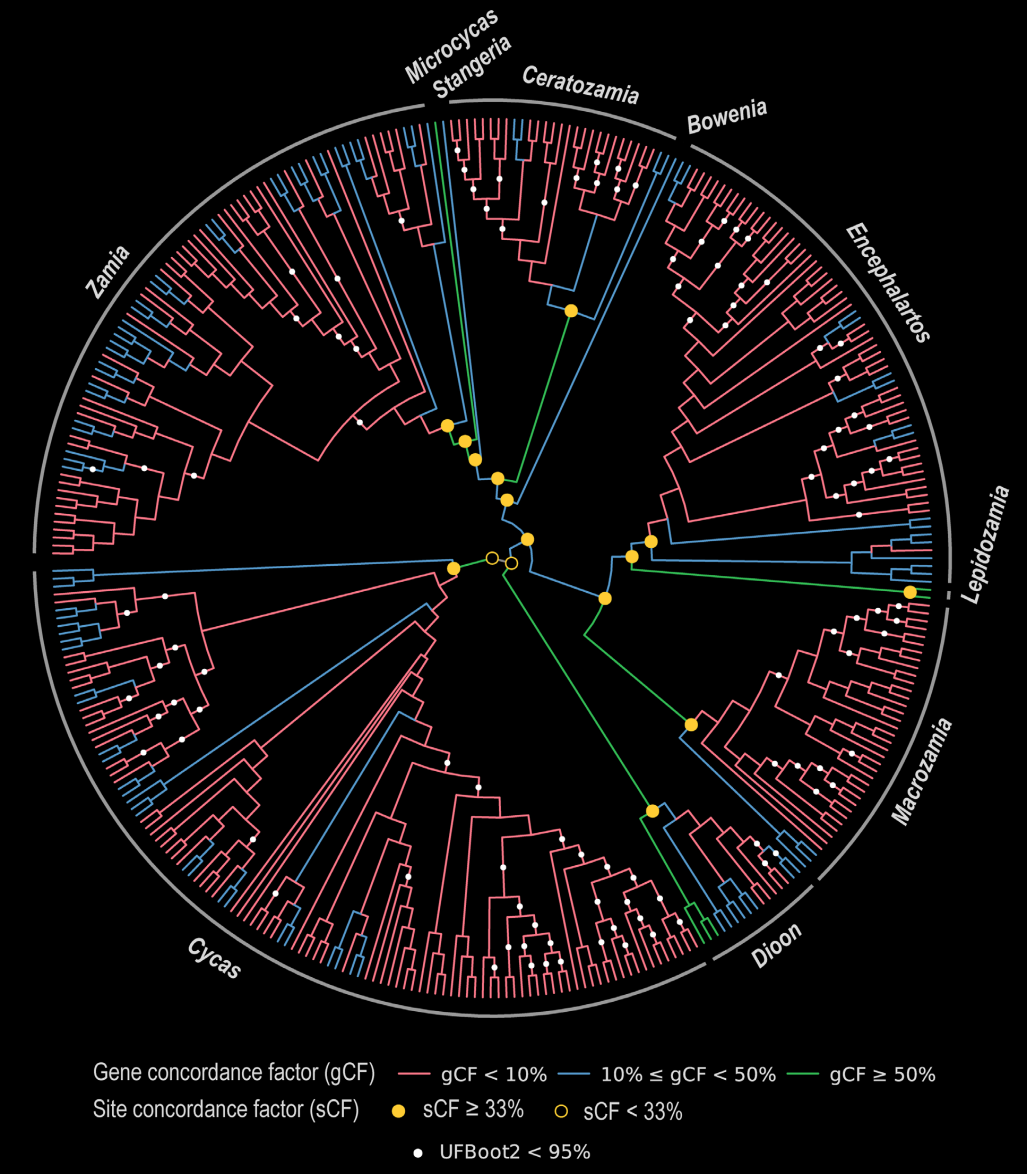
Radial cladogram of 346 cycad accessions plus the outgroup *Ginkgo biloba* (347 total). Branch color indicates gene concordance factor (gCF): red, gCF < 10%; blue, 10% ≤ gCF < 50%; green, gCF ≥ 50%. White solid circles on branches indicate ultrafast bootstrap (UFBoot2) support < 95% (unmarked branches have ≥ 95%). Gold circles at selected nodes indicate site concordance factor (sCF): filled if sCF ≥ 33%, open if sCF < 33% (33% represents the random expectation under equal quartet resolution frequencies). Concordance factors quantify genomic concordance with each branch (not statistical support); UFBoot2 quantifies resampling support under the concatenation model.

The partitioned maximum likelihood analysis recovered a well-supported phylogeny for all 346 cycad accessions (Fig. 1; Suppl. material 6). All 10 genera were monophyletic, with 100% bootstrap support and high crown-node gene concordance (gCF: 62–81%). Within Zamiaceae, *Dioon* was sister to the remaining genera, which formed two principal clades: one comprising *Encephalartos*, *Lepidozamia*, and *Macrozamia*, and the other comprising *Bowenia*, sister to *Stangeria*, *Ceratozamia*, *Microcycas*, and *Zamia*.

Ultrafast bootstrap support was high across the tree, with 240 of 344 scored internal branches (69.8%) receiving ≥95% support and a median bootstrap value of 100. Gene concordance factors were substantially lower: the median gCF was 3.2% (computed over a median of 1,315 decisive gene trees per branch), and 272 of 344 nodes (79.1%) had gCF below 10%. The dominant mode of gene tree discordance was polyphyly (mean gDFP = 89.0%) rather than support for alternative resolutions (mean gDF1 = 1.6%; mean gDF2 = 1.7%). Site concordance factors indicated a detectable site-level signal at most nodes, with a mean sCF of 40.3% and approximately 71% of scored nodes exceeding the 33.3% random expectation threshold. Per-branch concordance values are provided in Suppl. material 3.

Concordance and support among inter-generic backbone nodes were heterogeneous despite uniformly high bootstrap values. The *Microcycas*-*Zamia* sister relationship received the strongest genomic support (gCF = 66.7%, sCF = 85.5%), whereas the placements of *Bowenia* (gCF = 11.6%, sCF = 33.8%) and *Stangeria* (gCF = 16.1%, sCF = 36.8%) were only weakly supported, with site concordance near the random expectation threshold. The placement of *Bowenia* as sister to *Stangeria* + *Ceratozamia* + *Microcycas* + *Zamia* is congruent with Liu et al. (2022) and with the total-evidence analysis of Coiro et al. (2023) but differs from Salas-Leiva et al. (2013), who recovered *Bowenia* as sister to all remaining Zamiaceae excluding *Dioon*.

### Divergence time estimates

The timetree recovered a crown age of *ca*. 359 Ma (95% CI: 320–359 Ma) for the Cycadales, calibration-bound at the maximum constraint and consistent with the Carboniferous–Permian age inferred from total-evidence analyses of the fossil record. The Zamiaceae crown was estimated at *ca*. 194 Ma (95% CI: 165–196 Ma), within its calibration range (160–236 Ma). Backbone divergence times generally fell within their calibration ranges (28–66% of the allowed interval), indicating that these ages are informed by the molecular branch lengths rather than driven solely by the calibration bounds.

**Figure 2.**
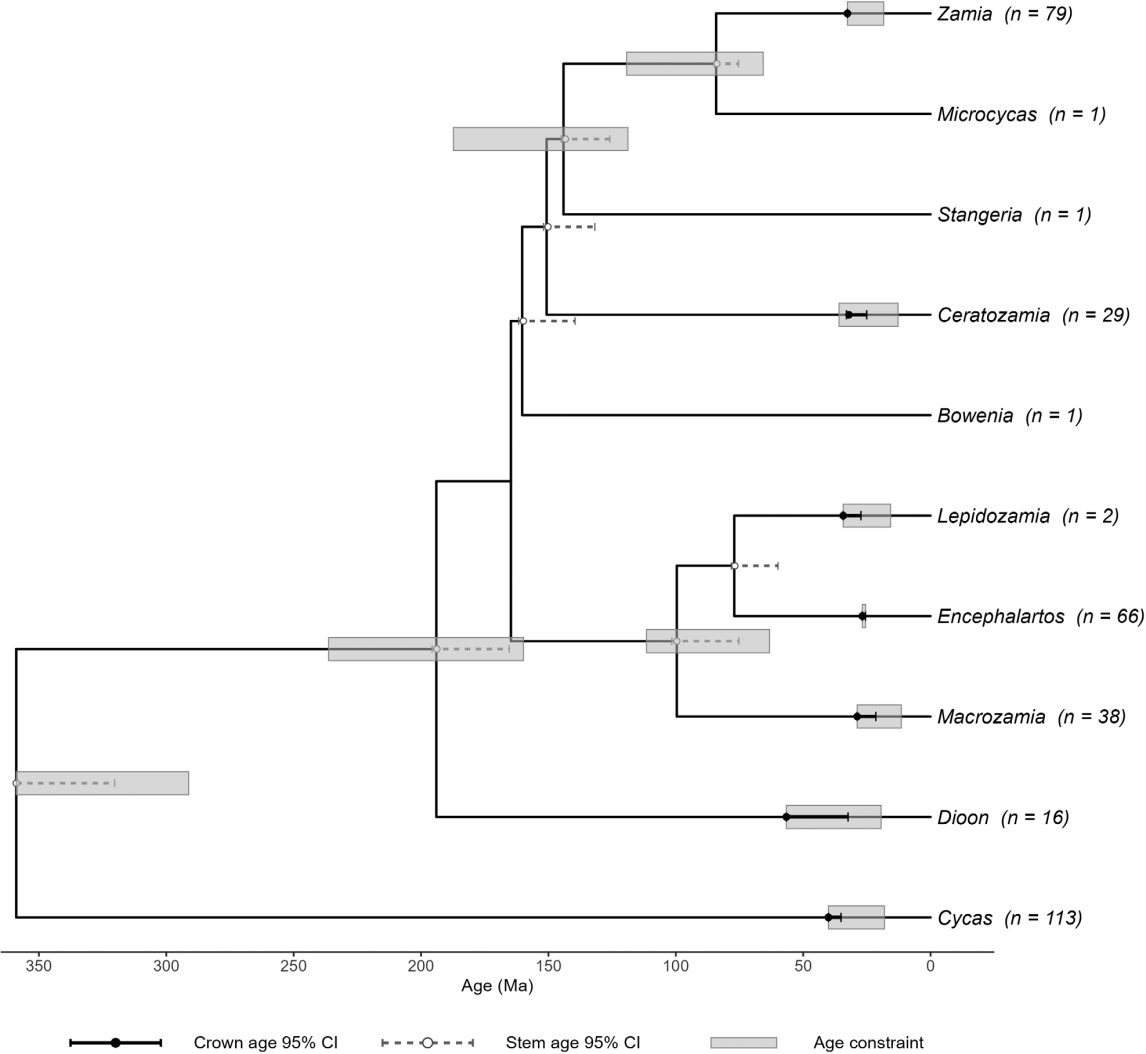
Genus-level summary of the cycad timetree, obtained by collapsing the full species-level timetree to one lineage per genus. Branch lengths are in millions of years (Ma). Counts (n) indicate the number of sampled taxa per genus in the full dataset. Filled black circles and solid black bars indicate genus crown ages and their 95% bootstrap confidence intervals; open grey circles and dashed grey bars indicate stem ages and their 95% bootstrap confidence intervals. Grey boxes indicate age constraints applied during divergence-time estimation.

Crown ages of genera were estimated in the Oligocene–Miocene but were predominantly calibration-bound; for six of the seven genera with crown calibrations, the estimated age converged on the calibration maximum (Fig. 2). These calibration-bound crown ages were: *Dioon* 57 Ma (95% CI: 32–57 Ma), *Cycas* 40 Ma (95% CI: 35–40 Ma), *Lepidozamia* 34 Ma (95% CI: 27–34 Ma), *Zamia* 33 Ma (95% CI collapsed to a single value, with all bootstrap replicates converging on the calibration maximum), *Macrozamia* 29 Ma (95% CI: 21–29 Ma), and *Encephalartos* 27 Ma (95% CI: 26–27 Ma). The sole exception was *Ceratozamia*, whose crown age of 32 Ma (95% CI: 25–33 Ma) fell within its calibration range (13–36 Ma), indicating that this estimate is informed by the molecular data. The mean 95% confidence interval width across all 345 internal nodes was 3.97 Ma. All node ages and confidence intervals are provided in Suppl. material 2.

## Discussion

### Interpreting high bootstrap support and low gene concordance

The combination of high bootstrap support (70% of nodes ≥95%) and low gene concordance factors (median gCF = 3.2%) is not unusual in phylogenomics because these metrics capture different aspects of support. Bootstrap values measure sampling support for the concatenated topology, whereas gCF measures agreement among individual gene trees and can remain low when loci are weakly informative or poorly resolved (Minh et al. 2020a; Lanfear and Hahn 2024). In cycads, the dominant mode of discordance is gene tree polyphyly (mean gDFP = 89%) rather than support for alternative resolutions, consistent with soft rather than hard incongruence; that is, insufficient information within individual loci rather than strong support for conflicting topologies (Wendel and Doyle 1998; Calonje et al. 2019).

This pattern is likely amplified by the slow molecular evolutionary rates characteristic of gymnosperms (De La Torre et al. 2017) together with recent rapid diversification within genera (Nagalingum et al. 2011). Individual loci therefore accumulate relatively few substitutions along short internal branches, leaving many gene trees weakly resolved even though concatenation across 1,409 loci yields strong cumulative support.

Site concordance factors remain above random expectation at most nodes (mean sCF = 40.3%; Mo et al. 2023), indicating detectable site-level signal even where gene tree concordance is low. Because sCF is computed via quartet sampling with three possible resolutions per branch, the expected value under no phylogenetic signal is 33.3%; a small number of backbone nodes approach this floor and should be interpreted with caution. This is particularly true for the branching order among *Bowenia*, *Stangeria*, and *Ceratozamia* within Zamiaceae, where all three inter-generic nodes have gCF below 20% and sCF values near 40% or lower. Although recent phylogenetic studies have recovered the same placement of *Bowenia* found here, as sister to the clade comprising *Stangeria*, *Ceratozamia*, *Microcycas*, and *Zamia* (Liu et al. 2022; Coiro et al. 2023), support for relationships in this part of the backbone remains weak.

Recent genus-level cycad phylotranscriptomic studies have likewise reported phylogenetic conflict, low quartet support, or limited concordance at some nodes. This includes in *Macrozamia* (Habib et al. 2022), *Ceratozamia* (Habib et al. 2023), *Zamia* (Lindstrom et al. 2024), *Encephalartos* (Habib et al. 2025), and *Dioon*, where Liu et al. (2026) found that fewer than 10% of gene trees supported many species-level relationships and attributed this discordance to rapid radiation and incomplete lineage sorting. Our results extend this pattern across all cycad genera, indicating that low gene-level concordance is a general feature of cycad phylogenomics rather than a genus-specific phenomenon.

This resource has several practical uses for cycad research. It provides a common species-level framework for evaluating taxonomic hypotheses, planning denser population-level sampling within genera, and interpreting trait, biogeographic, and diversification patterns in an explicit phylogenetic context. The dated tree also enables downstream conservation analyses based on phylogenetic diversity and evolutionary distinctiveness. Because the deposited trees and supporting files will be linked to the World List of Cycads, they should also provide a practical reference framework that can be updated as taxonomy and molecular sampling improve.

### Crown age estimates and calibration constraints

Crown ages for six of the seven calibrated genera fell at the maximum bound of their calibration constraints (Fig. 2, Table 1), indicating that these estimates are driven primarily by the calibrations rather than strongly informed by the molecular data alone. TreePL’s rate-smoothing penalty discourages abrupt rate variation across the tree; with the very short internal branches characteristic of recent generic radiations, this can push crown-age estimates toward older values so that substitutions are distributed more evenly across adjacent branches. By contrast, backbone divergence times, which are subtended by longer branches on both sides, are better constrained and occupy only 28–66% of their permitted calibration intervals. *Ceratozamia* is the sole exception among the calibrated crown ages: its estimated age (32 Ma) lies at 84% of its calibration interval (13–36 Ma), suggesting that molecular signal contributes more substantially to this estimate.

### Data products and intended use

The phylogram (maximum-likelihood tree including *Ginkgo biloba* as outgroup; Suppl. material 4) provides branch lengths in substitutions per site together with bootstrap support and concordance factors (gCF, sCF), allowing users to assess statistical support and genomic concordance across the tree. The timetree (cycads only, with *Ginkgo* pruned; Suppl. material 5) provides divergence time estimates in millions of years and 95% confidence intervals for all internal nodes. Both trees, together with the underlying gene trees, concatenated alignment, and associated metadata, are archived in Zenodo at https://doi.org/10.5281/zenodo.20074063.

Both trees are intended for use alongside the World List of Cycads (Calonje et al. 2026), with tip labels standardized to its accepted taxonomy so that tree tips can be linked directly to species records. Integration with the World List of Cycads also makes the phylogeny directly browsable in a web interface, allowing users without specialized phylogenetic software to quickly inspect relationships among species and clades. The downloadable tree files and associated data can also be utilized beyond the web interface, including for trait mapping and other comparative analyses. As taxonomy changes and new transcriptomic resources become available, the framework can be updated in future releases. The phylogeny and timetree presented here are best understood as current hypotheses that will be revised as new data become available.

## Conclusions

This study provides a densely sampled phylogeny and timetree for cycads spanning all 10 genera. The low gene concordance factors reported here, driven mainly by limited signal per locus rather than strong support for alternative topologies, show why concordance metrics should be reported alongside bootstrap support in phylogenomic studies of slowly evolving lineages. Together, these resources provide a framework for cycad systematics, comparative biology, and conservation analyses, including metrics such as phylogenetic diversity and evolutionary distinctiveness (e.g., EDGE scores; Isaac et al. 2007). All trees, gene trees, the concatenated alignment, and associated partition definitions, support values, confidence intervals, and calibration data are archived in Zenodo at https://doi.org/10.5281/zenodo.20074063 and linked to the World List of Cycads (Calonje et al. 2026).

## Additional information

### Artificial Intelligence (AI) use

The authors accept full responsibility for the content of the manuscript, including the disclosure of any use of AI.

Regarding the use of AI in the preparation of this manuscript, the authors declare the following: Claude, GPT; Used for: Language, style and writing, Software and automation.

Artificial-intelligence tools (Anthropic Claude Code, OpenAI ChatGPT) were used for limited assistance with manuscript editing and minor scripting tasks. All analyses, code, references, and scientific interpretations were reviewed and finalized by the authors.

### Author ORCIDs

M. Calonje https://orcid.org/0000-0001-9650-3136

J.A.R. Clugston https://orcid.org/0000-0002-3653-6953

M. Coiro https://orcid.org/0000-0002-0113-0320

### Data availability

All phylogenomic data supporting this study, including the concatenated alignment, partition definitions, phylogram, timetree, gene trees, calibration file, confidence-interval summary, and tip-label mapping, are archived in Zenodo at https://doi.org/10.5281/zenodo.20074063. SRA accession numbers for all transcriptome datasets are listed in Suppl. material 1. All other data that support the findings of this study are available in the main text and Supplementary materials.

## Supplementary materials

Supplementary material 1 Taxon sampling details for all 347 taxa Data type xlsx Explanation note **table SS1**: Taxon sampling details for all 347 taxa (346 cycads plus the outgroup *Ginkgo biloba*). Columns: current accepted species name, genus, NCBI accession number, data type (transcriptome or genome), tip label used in deposited trees, original name in SRA (where different), and renaming notes. https://binary.pensoft.net/file/1654554This dataset is made available under the Open Database License (http://opendatacommons.org/licenses/odbl/1.0/). The Open Database License (ODbL) is a license agreement intended to allow users to freely share, modify, and use this Dataset while maintaining this same freedom for others, provided that the original source and author(s) are credited. Michael Calonje, James A. R. Clugston, Mario Coiro

Supplementary material 2 Divergence time estimates and 95% bootstrap confidence intervals Data type xlsx Explanation note **table S2**: Divergence time estimates and 95% bootstrap confidence intervals for all 345 internal nodes of the cycad timetree. Columns: node identifier, node description, number of descendant tips, bootstrap support, median age (Ma), lower 2.5% CI (Ma), upper 97.5% CI (Ma), CI width (Ma), number of bootstrap replicates, and number of replicates recovering the node as monophyletic. https://binary.pensoft.net/file/1654555This dataset is made available under the Open Database License (http://opendatacommons.org/licenses/odbl/1.0/). The Open Database License (ODbL) is a license agreement intended to allow users to freely share, modify, and use this Dataset while maintaining this same freedom for others, provided that the original source and author(s) are credited. Michael Calonje, James A. R. Clugston, Mario Coiro

Supplementary material 3 Concordance factor summary for all 345 internal branches Data type xlsx Explanation note **table S3**: Concordance factor summary for all 345 internal branches. Columns: branch ID, bootstrap support, branch length, gene concordance factor (gCF), gene discordance factors (gDF1, gDF2), gene discordance due to polyphyly (gDFP), number of decisive gene trees (gN), site concordance factor (sCF), site discordance factors (sDF1, sDF2), and number of informative sites (sN). https://binary.pensoft.net/file/1654556This dataset is made available under the Open Database License (http://opendatacommons.org/licenses/odbl/1.0/). The Open Database License (ODbL) is a license agreement intended to allow users to freely share, modify, and use this Dataset while maintaining this same freedom for others, provided that the original source and author(s) are credited. Michael Calonje, James A. R. Clugston, Mario Coiro

Supplementary material 4 Phylogram Data type nexus Explanation note file S1: Phylogram (NEXUS format): maximum likelihood tree with 347 taxa, branch lengths in substitutions/site, node annotations for bootstrap, gCF, and sCF. https://binary.pensoft.net/file/1654557This dataset is made available under the Open Database License (http://opendatacommons.org/licenses/odbl/1.0/). The Open Database License (ODbL) is a license agreement intended to allow users to freely share, modify, and use this Dataset while maintaining this same freedom for others, provided that the original source and author(s) are credited. Michael Calonje, James A. R. Clugston, Mario Coiro

Supplementary material 5 Timetree Data type nexus Explanation note file S2: Timetree (NEXUS format): time-calibrated tree with 346 cycad accessions, branch lengths in millions of years, node annotations for bootstrap, gCF, sCF, median age, and 95% confidence intervals. https://binary.pensoft.net/file/1654558This dataset is made available under the Open Database License (http://opendatacommons.org/licenses/odbl/1.0/). The Open Database License (ODbL) is a license agreement intended to allow users to freely share, modify, and use this Dataset while maintaining this same freedom for others, provided that the original source and author(s) are credited. Michael Calonje, James A. R. Clugston, Mario Coiro

Supplementary material 6 Supplementary cladogram Data type pdf Explanation note file S3: Supplementary cladogram (PDF): rectangular cladogram of all 347 taxa (346 cycads plus the outgroup *Ginkgo biloba*) with bootstrap/gCF/sCF support values at each node and alternating genus shading for navigation. The 1,409 individual gene trees (combined_gene_trees.treefile, Newick format) are deposited with the Zenodo archive described in the Data availability section. https://binary.pensoft.net/file/1654559This dataset is made available under the Open Database License (http://opendatacommons.org/licenses/odbl/1.0/). The Open Database License (ODbL) is a license agreement intended to allow users to freely share, modify, and use this Dataset while maintaining this same freedom for others, provided that the original source and author(s) are credited. Michael Calonje, James A. R. Clugston, Mario Coiro
