# Supplementary figures and images for: A comprehensive phylogenomic framework for cycads (Cycadales)
Source: PhytoKeys. 2026 May 22;275:81–95. doi: 10.3897/phytokeys.275.194283 (PMC13221657; doi:10.3897/phytokeys.275.194283)

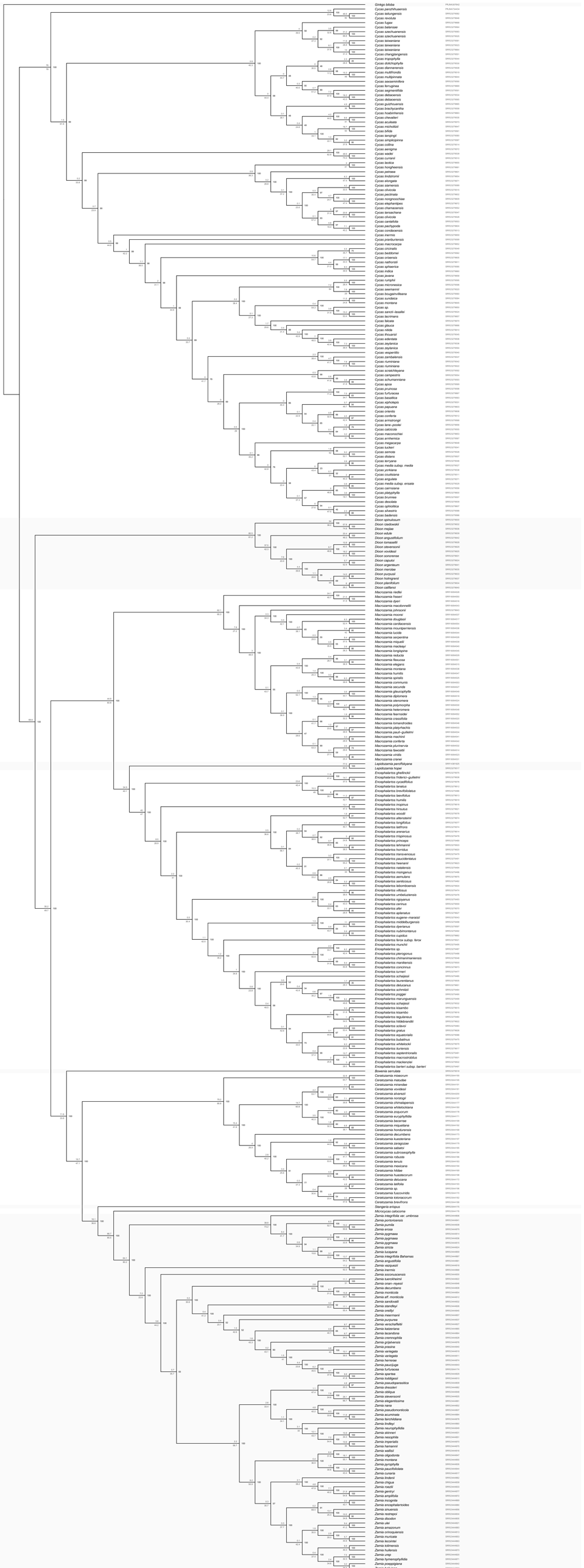

Supplement: Supplementary material 6 — Supplementary cladogram [file phytokeys-275-081_article-194283__-s006.pdf]
